# Procalcitonin Predicts Response to Beta-Lactam Treatment in Hospitalized Children with Community-Acquired Pneumonia

**DOI:** 10.1371/journal.pone.0036927

**Published:** 2012-05-17

**Authors:** Jérémie F. Cohen, Alexander Leis, Thibault Lecarpentier, Josette Raymond, Dominique Gendrel, Martin Chalumeau

**Affiliations:** 1 Department of Pediatrics, Saint-Vincent-de-Paul and Necker-Enfants-Malades Hospital, AP–HP, Université Paris-Descartes, Paris, France; 2 Epidemiological Research Unit on Perinatal Health and Women's and Children's Health (U953), Inserm, Paris, France; 3 Department of Microbiology, Cochin Hospital, AP–HP, Université Paris-Descartes, Paris, France; University of Tübingen, Germany

## Abstract

**Background:**

Antibiotic treatment of community-acquired pneumonia (CAP) in children remains mostly empirical because clinical and paraclinical findings poorly discriminate the principal causes of CAP. Fast response to beta-lactam treatment can be considered a proxy of pneumococcal aetiology. We aimed to identify the best biological predictor of response to beta-lactam therapy in children hospitalized for CAP.

**Methods:**

A retrospective, single-centre cohort study included all consecutive patients 1 month to 16 years old hospitalized in a teaching hospital in Paris, France, because of CAP empirically treated with a beta-lactam alone from 2003 to 2010. Uni- and multivariate analyses were used to study the ability of routine biological parameters available in the Emergency Department to predict a favourable response to beta-lactam (defined as apyrexia within 48 hours of treatment onset).

**Results:**

Among the 125 included patients, 85% (106) showed a favourable response to beta-lactam. In multivariate logistic regression, we found procalcitonin (PCT) the only independent predictor of apyrexia (p = 0.008). The adjusted odds ratio for the decadic logarithm of PCT was 4.3 (95% CI 1.5–12.7). At ≥3 ng/mL, PCT had 55.7% sensitivity (45.7–65.3), 78.9% specificity (54.4–93.9), 93.7% positive predictive value (84.5–98.2), 24.2% negative predictive value (14.2–36.7), 2.64 positive likelihood ratio (1.09–6.42) and 0.56 negative likelihood ratio (0.41–0.77). In the 4 children with a PCT level ≥3 ng/mL and who showed no response to beta-lactam treatment, secondary pleural effusion had developed in 3, and viral co-infection was documented in 1.

**Conclusions:**

PCT is the best independent biologic predictor of favourable response to beta-lactam therapy in children hospitalized for CAP. Thus, a high PCT level is highly suggestive of pneumococcal aetiology. However, a 3-ng/mL cut-off does not seem compatible with daily medical practice, and additional research is needed to further define the role of PCT in managing CAP in children.

## Introduction

Antibiotic treatment of community-acquired pneumonia (CAP) in children remains mostly empirical because determining the aetiologic pathogen is difficult in this age group. Clinical findings [1], chest radiography [2], routine blood analyses (blood cell count, C-reactive protein [CRP] level) or rapid urinary antigen detection tests [3] poorly discriminate the principal causes of CAP (*Streptococcus pneumoniae*, *Mycoplasma pneumoniae* and respiratory viruses). Blood culture results are available only after 2 or 3 days and are positive in only <10% of authentic bacterial CAP. European and American guidelines therefore recommend as the first choice empirical beta-lactam treatment to target *S. pneumoniae* in hospitalized children of all ages [4]–[8]. In 95% of children with bacteraemic pneumococcal CAP, apyrexia is reached within 48 hr of antibiotic treatment [9], and thus, clinical response to beta-lactam treatment can be considered a proxy of pneumococcal aetiology.

To date, no biologic predictor of clinical response to beta-lactam treatment has been found. We aimed to identify predictors of clinical response among routine biochemical markers available at admission of children hospitalized with CAP.

## Methods

### Study design

We conducted a retrospective single-centre hospital-based cohort study in a teaching hospital in Paris, France. We analyzed data from all consecutive children aged 1 month to 16 years who were hospitalized between January 2003 and July 2010 for CAP and empirically treated with beta-lactam monotherapy (amoxicillin, 80–100 mg/kg/day, or ceftriaxone, 50 mg/kg/day). As in previous studies, CAP was defined as the association of fever (temperature ≥38°C), respiratory symptoms and pulmonary condensation on chest radiography [10], [11]. We excluded children who had received antibiotics in the 10 days before admission, those with an underlying chronic respiratory disease except asthma (bronchial ectasia, bronchopulmonary dysplasia, cystic fibrosis), and those with a chronic neurologic disease or a known immunodeficiency syndrome. Children with empyema or pleural effusion at the time of CAP diagnosis were excluded because these patients usually show a delayed response to antibiotherapy [12], [13] but children developing pleural effusion during hospitalization were kept for analysis. During the study period, the local protocol was to prescribe oral amoxicillin as first-line treatment for children hospitalized with CAP and intravenous amoxicillin or ceftriaxone when there were concerns about oral absorption (e.g., because of vomiting).

### Outcome

The outcome was clinical response to beta-lactam treatment, defined as apyrexia within the first 48 hr of treatment (success) or after 48 hr of treatment (failure) [7], [8]. During the study period, all children underwent axillary temperature measurement every 6 hr. Temperature was abstracted from clinical charts by one author blinded to all potential predictors, and apyrexia was defined as 3 consecutive measurements of temperature <38°C. During the study period, according to the local protocol, patients were discharged after 3 consecutive temperature measurements <38°C.

### Potential predictors

During the study period, the local routine protocol for the work-up of children hospitalized with CAP included, at the time of arrival in the emergency department, complete blood count (Coulter counter), CRP level (nephelometry), and procalcitonin level (PCT; immunoluminometric assay, LUMItest, Brahms Diagnostica, Berlin, Germany). The manufacturer's detection threshold for the PCT assay was 0.1 ng/mL.

### Microbiology

Nonsystematic microbiological procedures available during the study period included blood culture with antibiogram and minimum inhibitory concentration (MIC) assessment before first administration of antibiotics, nasopharyngeal swab for fluorescent viral immunoassay (respiratory syncytial virus [RSV]; influenza virus A and B; parainfluenza virus 1, 2 and 3; human metapneumovirus [HMPV] and adenovirus), bocavirus and *M. pneumoniae*-specific polymerase chain reaction (PCR) assay. When pleural effusion appeared during CAP, pleural fluid was obtained by pleural puncture or surgical drainage and sent for bacterial analyses (microscopy direct examination, bacterial culture, *S. pneumoniae* antigen detection immunoassay [Binax NOW, Inverness Medical, Scarborough, ME] and a specific *S. pneumoniae* PCR).

### Statistical analysis

We first described the study population characteristics, including the identified pathogens. Values for potential predictors were compared by Mann-Whitney *U* test according to clinical response to beta-lactam treatment. Independent predictors were identified after adjustment on a multivariate logistic model. In case of strong correlation between 2 variables (*r*>0.50, Pearson pairwise correlation test), we retained the variable with fewer missing data. When the relationship between clinical response to beta-lactam treatment and continuous potential predictors was not linear (chi-square test on deviance difference), continuous variables were transformed into fractional polynomials of as low degree as possible [14], and the final model selection involved the Royston & Altman's algorithm [15]. The goodness of fit of the model was assessed with a Hosmer-Lemeshow test [16]. We performed sensitivity analyses to study the potential effect of the selection of variables on the main results of the study. For the best identified predictor on multivariate analysis, we analyzed diagnostic performance (sensitivity, specificity, likelihood ratios and predictive values) at different rounded thresholds. For each threshold, sensitivity was defined as the proportion of patients with a value for a predictor above or equal to the given threshold among those with clinical response to beta-lactam. Statistical analyses involved use of Stata/SE 10 (StataCorp, College Station, TX, USA).

### Ethics

The Ethical Review Committee “Comité de Protection des Personnes Ile de France III” has examined the research which was found to conform to generally accepted scientific principles and research ethical standards and to be in conformity with the laws and regulations of the country in which it was performed (File S1). The waiver of consent was approved by the Institutional Review Committee (Comité de Protection des Personnes Ile de France III).

## Results

### Population characteristics and microbiology results

We identified 125 children who met the inclusion criteria. The mean age (SD) of children was 3.1 years (2.7), 70 were girls and 24 (19%) had asthma. At least one pathogen was identified in 47/123 (38%) of cases. We found 8/87 (9%) positive blood cultures for *S. pneumoniae*, and all 8 strains were penicillin sensitive (MIC<0.06 µg/mL). All patients with positive blood culture results for *S. pneumoniae* showed apyrexia within 48 hr of treatment onset. These patients had higher PCT levels than those with negative blood culture results (median 9.4 vs. 3.2 ng/mL, p = 0.06, Mann-Whitney *U* test). PCR results for *M. pneumoniae* were positive in 5/64 (8%) of cases, and nasopharyngeal swab results were positive for viruses in 35/109 (32%) of cases, with RSV found in 17/35 (49%) cases, influenza A in 9/35 (26%) cases, adenovirus in 3/35 (9%) cases, enterovirus in 2/35 (6%) cases, and influenza B, parainfluenza 3, HMPV and bocavirus in 1/35 (3%) cases each. The patient with a positive bocavirus PCR was admitted on day 4 of fever and then showed apyrexia at the end of day 3 of beta-lactam treatment. *S. pneumoniae* co-infection was suspected in this patient on the basis of a positive urinary antigen test result. In 3 children, secondary pleural effusion developed during hospitalization; 2 underwent surgical pleural drainage and 1 ultrasound-guided pleural puncture. In 2 of these 3 children, pleural fluid results remained negative, but the third case had positive results on *S. pneumoniae* antigen detection immunoassay of pleural fluid.

### Clinical response to beta-lactam treatment

Most of the children, 106/125 (85%), showed clinical response to beta-lactam treatment. Among the 19 other patients, 4 received macrolide treatment after the first 48 hr of beta-lactam treatment and then showed stable apyrexia within 48 more hours. Two of them had positive PCR results for *M. pneumoniae* (among 10 patients tested for *M. pneumoniae* by PCR). In all, only 1/125 patients was readmitted in the same hospital within 15 days after discharge because of febrile relapse. This patient was considered a non-responder in the analyses. Among 125 patients, 38 (30%) received oral amoxicillin, 26 (21%) intravenous amoxicillin and 61 (49%) intravenous ceftriaxone. Response or not to antibiotic treatment did not differ by antibiotic (amoxicillin vs. ceftriaxone, p = 0.90) or route of administration (oral vs. intravenous, p = 0.51). Children with a response or not to beta-lactam treatment did not differ significantly in age (median 2.7 vs. 2.2 years, p = 0.43) or prevalence of asthma (19.8% vs. 15.8%, p = 0.68).

### Prediction of clinical response to beta-lactam treatment

The median neutrophil count and PCT level were significantly higher in children with than without a response to beta-lactam treatment (14790 vs. 12330/mm^3^ and 3.7 vs. 0.7 ng/mL, respectively, p<0.05, Table 1). The median white blood cell (WBC) count and CRP level were higher but not significantly in children with than without a treatment response (18850 vs. 15400/mm^3^, p = 0.06; 155 vs. 59 mg/L, p = 0.13, respectively). The WBC and neutrophil counts were highly correlated (*r* = 0.92, p<0.001). Neutrophil count contained several missing data (22/125; 18%), so neutrophil count was excluded from the multivariate logistic model. Because the relationship between clinical response to beta-lactam treatment and PCT level as a continuous variable was non-linear (p = 0.02), PCT level was transformed into a first-degree fractional polynomial by natural logarithmic transformation. No transformation was needed for CRP level or WBC count (p = 0.87 and p = 0.16 respectively). After adjustment for CRP level, WBC and PCT level in a logistic model, only PCT remained independently and significantly associated with clinical response to beta-lactam treatment (p = 0.008). The adjusted odds ratio for the decadic logarithm of PCT was 4.3 (95% CI 1.5–12.7). Every 10-fold increase in PCT level was associated with 4.3-fold odds of beta-lactam treatment being successful. The fit of the logistic model was good (p = 0.67). We conducted a sensitivity analysis to evaluate the potential effect of not including age in the multivariate model and found no change in the main results of the study: age as a dichotomous variable (<2 vs. ≥2 yr) or a continuous variable after transformation into fractional polynomials was not associated with response to antibiotics treatment (p = 0.66 for both), and PCT remained the only predictor of success after adjustment for age. We also conducted a sensitivity analysis that included neutrophil count instead of WBC count in the multivariate model. The main results of the study were stable: PCT remained the only predictor associated with response to antibiotics treatment, with a slight increase in the adjusted odds ratio (from 4.3 [1.5–12.7] to 5.4 [1.6–17.5]).

**Table 1:** Analysis of predictors according to clinical response to beta-lactam treatment.

		Apyrexia within 48 hr of treatment			Apyrexia after 48 hr of treatment			
	N	Mean	Median	IQR	Mean	Median	IQR	*p*
WBC count (/mm^3^)	125	20275	18850	12700–26900	15179	15400	9800–19600	0.06
Neutrophil count (/mm^3^)	103	15941	14790	8610–21740	10966	12330	7200–16380	0.04
CRP level (mg/L)	125	163	155	51–242	119	59	27–266	0.13
PCT level (ng/mL)	125	8.7	3.7	1–9.4	3.1	0.7	0.2–2.9	0.002

Abbreviations: WBC, white blood cell; CRP, C-reactive protein; PCT, procalcitonin; IQR, interquartile range.

### PCT for the prediction of apyrexia

**Figure 1:**
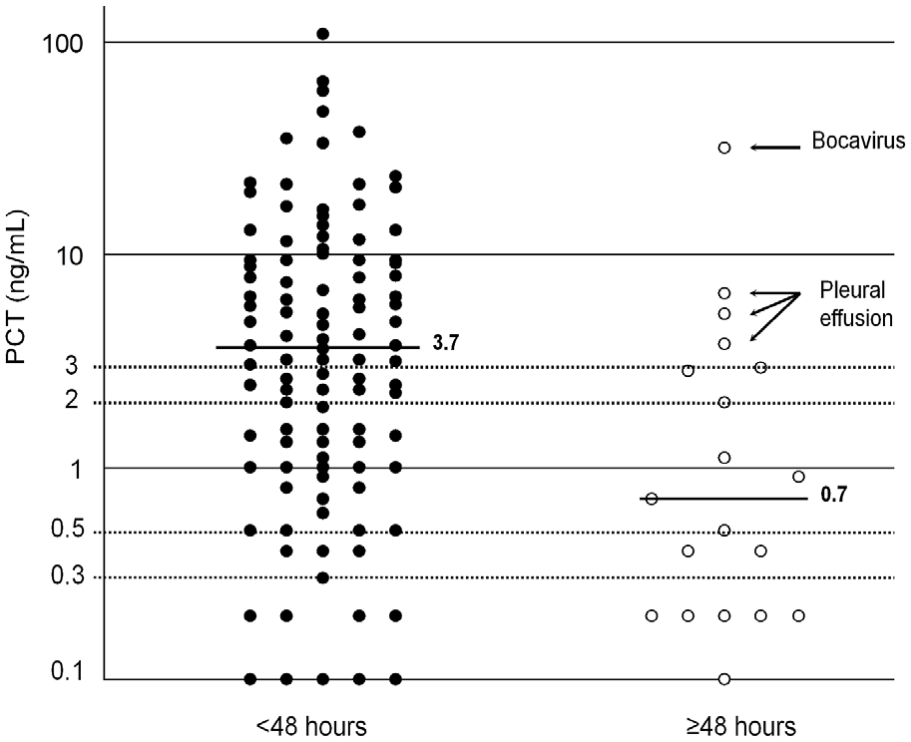
Distribution of procalcitonin (PCT) level according to clinical response to beta-lactam treatment. The short, bold, horizontal lines are the median for each group.

The detailed performance of PCT level in predicting apyrexia is in Table 2. Among 63 children with a PCT level ≥3 ng/mL, 59 (94%) showed apyrexia within the first 48 hr of treatment and 47 (75%) within the first 24 hr. Among the 4 patients with a PCT level ≥3 ng/mL who showed no response to beta-lactam treatment, secondary pleural effusion developed in 3, and bocavirus infection mixed with suspected *S. pneumoniae* infection (positive urinary antigen detection test) was documented in 1 (Figure 1). With a 3-ng/mL cutoff, the performance of PCT was 55.7 (95% CI 45.7–65.3) and 78.9% (54.4–93.9) for sensitivity and specificity, respectively; 93.7% (84.5–98.2) and 24.2 (14.2–36.7) for positive and negative predictive values, respectively; 2.64 (1.09–6.42) and 0.56 (0.41–0.77) for positive and negative likelihood ratio, respectively; and 4.7 (1.5–14.4) odds ratio.

**Table 2:** Predictive performance of procalcitonin (PCT) level in clinical response to beta-lactam treatment.

PCT	Sensitivity (%)	Specificity (%)	PPV (%)	NPV (%)	PLR	NLR
0.3	91.5 [84.5–96.0]	31.6 [12.6–56.6]	88.2 [80.6–93.6]	40.0 [16.3–67.7]	1.34 [0.98–1.83]	0.27 [0.11–0.67]
0.5	87.7 [79.9–93.3]	42.1 [20.3–66.5]	89.4 [81.9–94.6]	38.1 [18.1–61.6]	1.52 [1.03–2.24]	0.29 [0.14–0.61]
1	79.2 [70.3–86.5]	57.9 [33.5–79.7]	91.3 [83.6–96.2]	33.3 [18.0–51.8]	1.88 [1.10–3.22]	0.36 [0.21–0.61]
2	65.1 [55.2–74.1]	63.2 [38.4–83.7]	90.8 [81.9–96.2]	24.5 [13.3–38.9]	1.77 [0.96–3.24]	0.55 [0.36–0.85]
3	55.7 [45.7–65.3]	78.9 [54.4–93.9]	93.7 [84.5–98.2]	24.2 [14.2–36.7]	2.64 [1.09–6.42]	0.56 [0.41–0.77]
4	48.1 [38.3–58.0]	84.2 [60.4–96.6]	94.4 [84.6–98.8]	22.5 [13.5–34.0]	3.05 [1.06–8.77]	0.62 [0.47–0.81]
5	42.5 [32.9–52.4]	84.2 [60.4–96.6]	93.8 [82.8–98.7]	20.8 [12.4–31.5]	2.69 [0.93–7.77]	0.68 [0.53–0.88]
10	23.6 [15.9–32.8]	94.7 [74.0–99.9]	96.2 [80.4–99.9]	18.2 [11.1–27.2]	4.48 [0.65–31.13]	0.81 [0.69–0.94]

PCT thresholds are in ng/mL.

Numbers in square brackets are 95% confidence intervals.

Abbreviations: PPV, positive predictive value; NPV, negative predictive value; PLR, positive likelihood ratio, NLR, negative likelihood ratio.

## Discussion

This is the first study aiming to identify biological predictors of clinical response to antibiotic treatment in children hospitalized with CAP. As compared with WBC count, neutrophil count and CRP level, PCT level was the best marker routinely tested that predicted apyrexia. These results are consistent with those of 5 of 7 studies investigating the ability of PCT level to distinguish the different aetiologic pathogens of CAP in children [11], [17]–[22]. In our study, 94% of children with a PCT level ≥3 ng/mL showed apyrexia within the first 48 hr of beta-lactam treatment, and 75% apyrexia within the first 24 hr of treatment. In 3 of the 4 children with a PCT level ≥3 ng/mL who showed no response to beta-lactam treatment, secondary pleural effusion had developed and in the other one, bocavirus infection was documented. A 3-ng/mL cutoff for PCT provided high specificity, positive predictive value and odds ratio. These results confirm the potential usefulness of PCT in estimating the efficacy of the pneumococcal conjugate vaccine among children [23] and determining the eligibility of children for clinical trials of CAP as has been proposed for adults [3], [24]. A recent study showed that a PCT-based approach could reduce antibiotic consumption in children with CAP [25]. Though our results suggest that a high PCT level is associated with pneumococcal aetiology, the likelihood ratios that we found for PCT were insufficient for everyday medical decision making and further investigation is needed to develop a PCT-based clinical decision rule. The PCT cut-off of ≥3 ng/ml should not be considered a threshold to identify pneumococcal CAP in daily clinical practice. Lower operational thresholds were reported in studies of adults [26] and children [25]. However, a PCT cut-off of ≥3 ng/ml is probably specific enough to be used as an eligibility criterion in studies estimating the efficacy of the pneumococcal conjugate vaccine in children [23] and for clinical trials of antibiotics in CAP [24].

Clinical response to beta-lactam treatment (i.e., reaching or not apyrexia within the first 48 hr of antimicrobial treatment) seemed an interesting outcome to predict because persistence of fever after 48 hr of an adapted antibiotic treatment is usually considered a failure and leads to new investigations (e.g., new chest radiography) and/or to antibiotherapy modification. Predicting a fast response to beta-lactam therapy might be considered a proxy to predict *S. pneumonia* as a causative pathogen in CAP. Toikka *et al.* showed that among 85 children with blood-culture–proven pneumococcal CAP, most (95%) showed apyrexia within the first 48 hr of antibiotic treatment [9]. In this Finnish study, as in ours, patients with no response to antibiotic treatment within 48 hr showed pleural effusion developed or an underlying chronic condition (chronic encephalopathy or respiratory disease). In another of our studies, most children (92%) with documented pneumococcal CAP showed defervescence within 24 h with beta-lactam monotherapy [10]. We considered clinical response to antibiotherapy within 48 hr of treatment onset a proxy for pneumococcal aetiology, but viral infections may resolve during the same time. We obtained only indirect evidence of the aetiology of CAP in most patients, and our results should be considered with caution. The fact that children with pneumococcal CAP mostly become afebrile within 48 hr of antibiotic treatment only indirectly supports the conclusion that most patients with CAP who became afebrile within 48 hr actually had pneumococcal infection.

The main limitation to this study is its design as a retrospective analysis, which could suggest loss of accuracy in data collection, especially clinical characteristics (e.g., apyrexia) and missing data (as observed for 18% of the neutrophil count). Furthermore, we defined CAP by the association of fever, respiratory symptoms and condensation on chest radiography, but chest radiographs were interpreted by only a single radiologist, so we were not able to estimate any inter-rater reliability. These non-differential biases and the absence of an *a priori* sample size calculation could have led to loss of power, but we were still able to find several statistically significant differences.

This study included children hospitalized because of the severity of their clinical features or because of their young age (mean age: 3.1 vs. median 4.1 years in published series in the literature) [10], [27]–[33]. This lower age could have resulted in an over-representation of viral CAP and an under-representation of *M. pneumoniae* CAP. The consequences of this selection bias cannot be precisely estimated because published epidemiological series of CAP in children show strong heterogeneity (different epidemiological contexts, different microbiological investigation methods). Likewise, we studied only children with severe CAP requiring hospitalization. We did not include children who had complicated CAP (i.e., empyema or pleural effusion on admission) or children with underlying chronic respiratory disease (except asthma). Further clinical research is needed to confirm that our findings also apply to these particular populations.

Another limitation of this study is that selection bias could have occurred because we included children hospitalized for CAP and empirically treated with a beta-lactam monotherapy during the first 48 hr for suspected *S. pneumoniae* infection. Indeed, in our study, the treating physician in charge of the patient in the emergency department was free to choose the antibiotherapy that he felt to be the most adapted to the patient's condition on the basis of implicit epidemiologic, clinical and paraclinical findings, including PCT level. This bias limits the reproducibility of our results to another population, because of the single-centre design of our study.

In conclusion, PCT level was found the only predictor of the clinical response to beta-lactam treatment in children hospitalized for CAP. Our data indirectly shows that a serum level of PCT ≥3 ng/mL is suggestive of pneumococcal CAP, but this threshold seems too high to be implemented in daily clinical practice to identify pneumococcal CAP. However, this threshold might be useful in epidemiological surveillance studies and clinical trials for CAP in children.
